# Development of a biaxial tensile testing machine for pulsed neutron experiments

**DOI:** 10.1016/j.mex.2019.08.015

**Published:** 2019-09-18

**Authors:** Koji Kiriyama, Shuoyuan Zhang, Hirotoshi Hayashida, Jun-ichi Suzuki, Toshihiko Kuwabara

**Affiliations:** a Comprehensive Research Organization for Science and Society (CROSS), Japan; b Tokyo University of Agriculture and Technology, Japan

**Keywords:** A biaxial tensile testing machine for pulsed neutron experiments (BTM-NEU), Biaxial tensile test, ISO standard, J-PARC, MLF, Pulsed neutron, Bragg-edge imaging

## Abstract

A biaxial tensile testing method has been used to get macroscopic information on elastoplastic deformation of a thin steel specimen and improve the accuracy of plastic processing of steel materials. We newly developed a biaxial tensile testing machine for pulsed neutron experiments (BTM-NEU) to provide the microscopic crystallographic information of steel materials under biaxial load and correlate it with the macroscopic mechanical properties of the materials. The performance of the BTM-NEU was experimentally evaluated with cold-rolled mild steel and hot-rolled high-tensile-strength steel materials and compared with that of a standard biaxial tensile testing machine (BTM-std) as follows.

• The BTM-NEU can test an ISO-standardized cruciform specimen as the BTM-std and its performance is equivalent to that of the BTM-std.
• The BTM-NEU has excellent long-time reliability and stability necessary for pulsed neutron experiments, especially Bragg-edge neutron imaging experiments.
• The BTM-NEU can be applied to pulsed neutron experiments using a Bragg-edge imaging method.

## Graphical abstract

**Figure:**
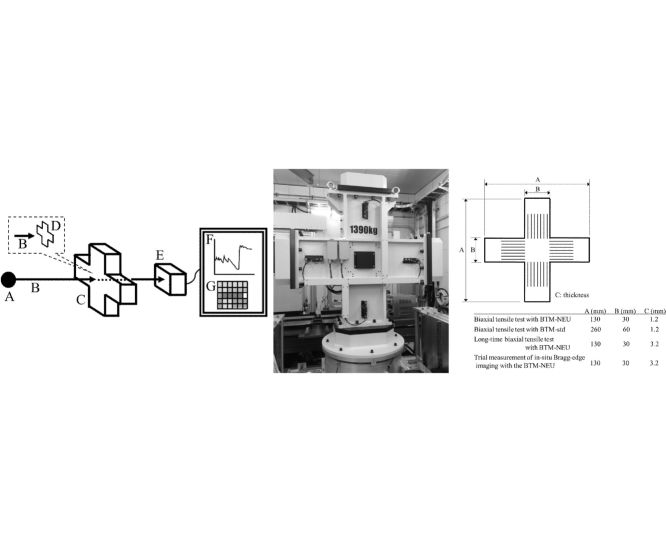

**Specification Table**

**Table:** 

Subject Area:	Engineering
More specific subject area:	Plastic processing
Method name:	A biaxial tensile testing machine for pulsed neutron experiments (BTM-NEU)
Name and reference of original method:	Name: Servo-controlled biaxial tensile testing machine. Reference: T. Kuwabara, S. Ikeda, K. Kuroda, Measurement and analysis of differential work hardening in cold-rolled steel sheet under biaxial tension, J. Mater. Process. Technol., 80-81 (1998), 517-523.
Resource availability:	None

## Method details

### Concept

To utilize highly functional thin steels for modern transportation equipment (e.g., automobiles), reliable press forming is needed. A biaxial tensile testing machine [[1], [2], [3], [4]], which equips with equi-displacement (pentagraph-type) mechanism [5,6] and two pairs of servo-controlled opposing hydraulic cylinders, was developed by the Kuwabara Laboratory in Tokyo University of Agriculture and Technology to provide experimental information on elastoplastic deformation of a cruciform thin steel specimen with a shape specified in the ISO standard [7]. We used the machine as a reference of our development. Therefore, we call it a standard biaxial tensile testing machine (BTM-std) in this paper. The machine has greatly contributed to obtain mechanical parameters of the specimen essential for the simulation of press forming and improve the accuracy in the simulation of press forming limit and deformation amount. As a result, press forming of products with complicated shapes has become possible. On the other hand, many researches on crystal plasticity models and microscopic states (e.g., internal stress and phase distribution among particles) using X-ray and neutron beams have been conducted to examine the deformation mechanism of steel materials under press forming. Recently, various crystal plasticity models have been proposed to predict various deformation behaviors. For the prediction of differential work-hardening behavior of steel sheets under biaxial tension, it has become clear that the consideration of slip systems in the models is important [8]. An X-ray diffraction method is one of the methods which can provide experimental information on the microscopic state of a cruciform steel specimen under biaxial load. However, the measuring area in the central part of the specimen is usually small and thin (e.g., 6 mm in diameter and 0.4 mm in thickness) to achieve a high load even with an intense synchrotron radiation beam [9,10]. In contrast, a neutron beam diffraction method has been used to study microscopic states (e.g., lattice strains relating to reciprocal lattice planes) in steel specimens with several mm and more in thickness [11] and the specimens under biaxial load [12,13]. However, ISO-standardized cruciform specimens, of which homogeneous large strain can be applied in the central part, had not been used in the studies. In recent years the Energy Resolved Neutron Imaging System (RADEN) was developed in the Materials and Life Science Experimental Facility (MLF) of the Japan Proton Accelerator Research Complex (J-PARC) [14]. RADEN is the world's first system dedicated to pulsed neutron imaging with a neutron beam with the largest beam cross section of 300 mm × 300 mm. It has become possible to provide spatial distribution of crystallographic information (e.g., lattice strain, phase, texture, crystal size, and dislocation density) in steel specimens with the large area of e.g. 30 mm × 30 mm by a single Bragg-edge imaging measurement [15].

**Fig. 1:**
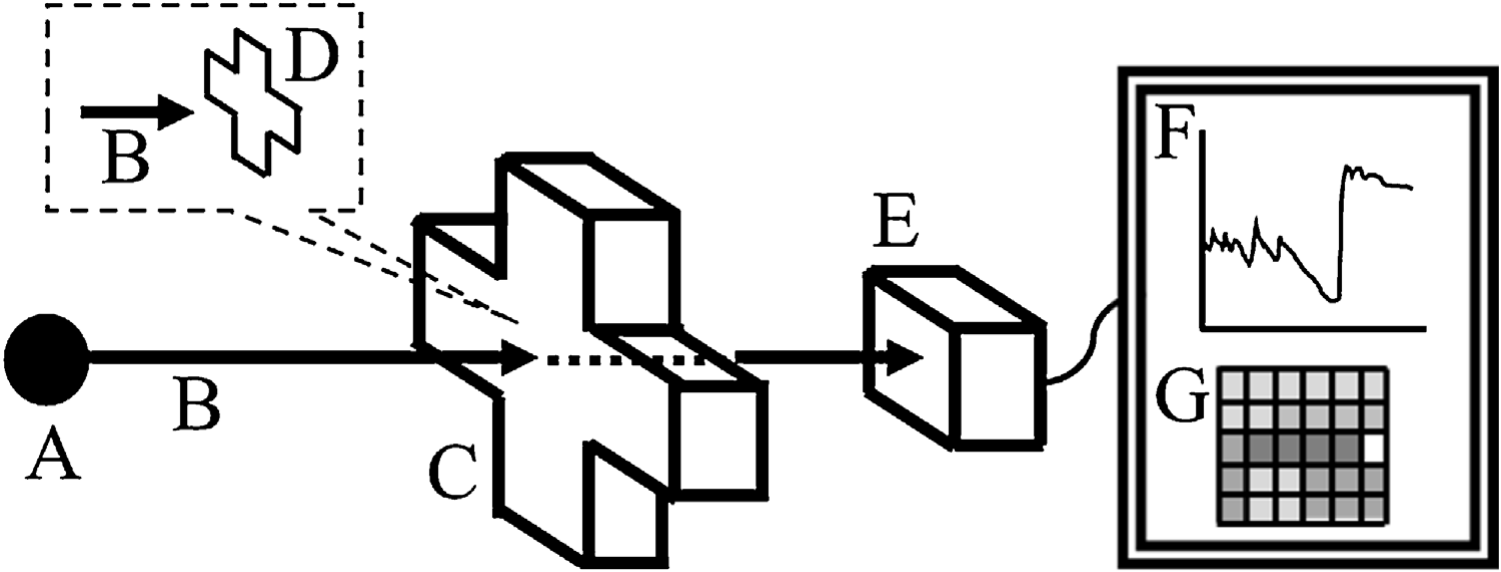
Conceptual diagram of a pulsed neutron imaging experiment with the BTM-NEU. A pulsed neutron beam (B) generated in the neutron source (A) of the MLF, J-PARC irradiates a cruciform specimen (D) placed in the BTM-NEU (C). The neutron beam transmitted through the specimen is measured with a two-dimensional neutron detector (E). A neutron transmission spectrum (F) containing the crystallographic information of each position of the specimen is analyzed by the Bragg-edge imaging method. Finally, a distribution diagram of the crystallographic information of the specimen (G) is obtained.

To perform neutron experiments on steel specimens under biaxial load with the RADEN, we developed a biaxial tensile testing machine for pulsed neutron experiments (BTM-NEU). The BTM-NEU is designed based on the design of the BTM-std which can test ISO-standardized cruciform specimens. Moreover, the BTM-NEU is designed to be placed vertically for neutron experiments, while the BTM-std is placed horizontally. Fig. 1 shows a conceptual diagram of a pulsed neutron imaging experiment with the BTM-NEU.

In this report, it is also shown that the performance of the BTM-NEU is experimentally verified to be equivalent to that of the BTM-std. Moreover, in Bragg-edge neutron imaging experiments, it is sometimes needed to measure a neutron transmission spectrum for a longer time than 10 h with the current RADEN. The stability of the BTM-NEU is also evaluated by a long-time biaxial tensile test and a trial measurement of in-situ Bragg-edge neutron imaging with the BTM-NEU is performed.

### Specifications of the BTM-NEU

**Fig. 2:**
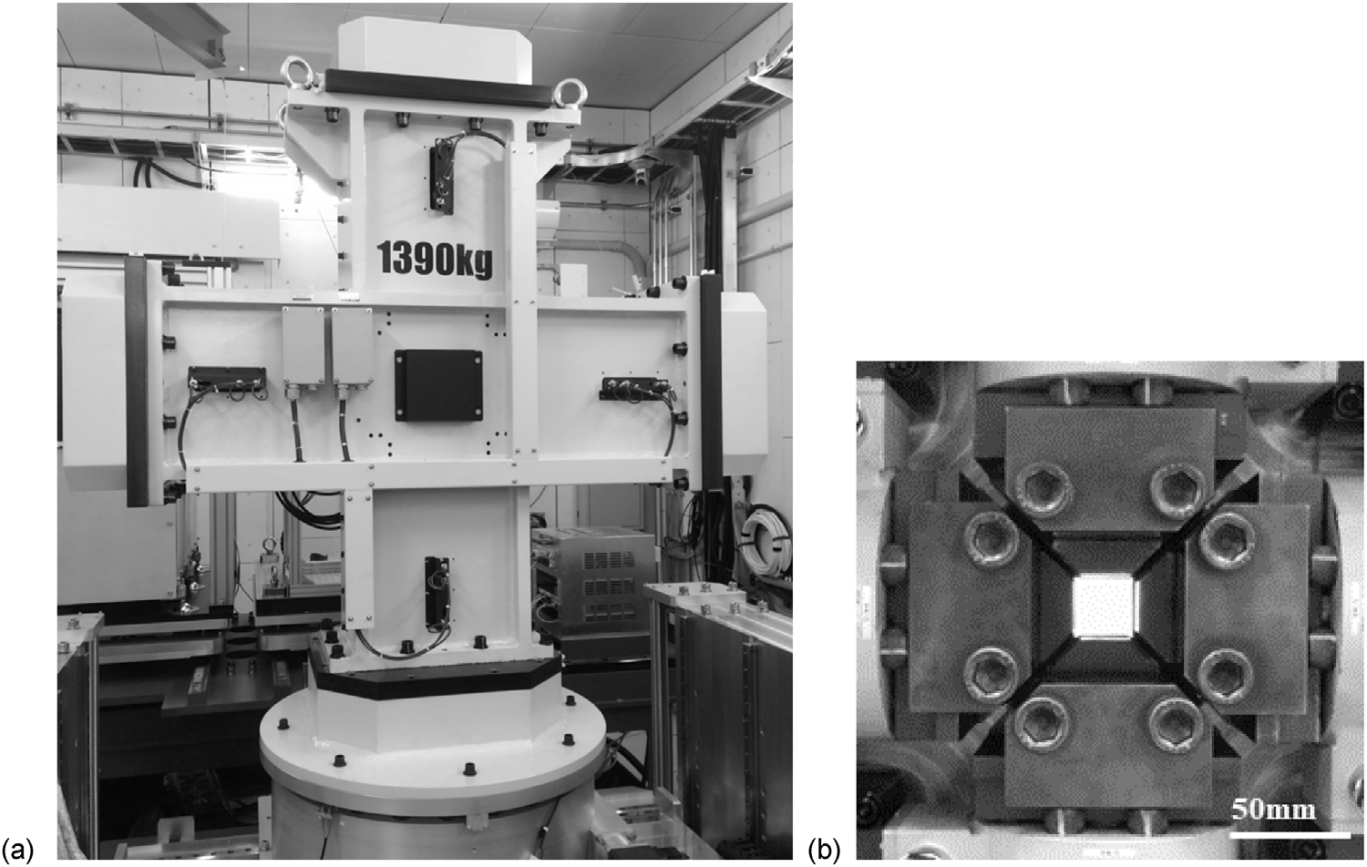
The biaxial tensile testing machine for pulsed neutron experiments (BTM-NEU) installed on a sample stage of the Energy Resolved Neutron Imaging System (RADEN) in the MLF, J-PARC, (b) Clamps placed in the center of the BTM-NEU. A cruciform specimen is fixed by the clamps. The white square area of the picture is an aperture for the passing of a neutron beam.

The pictures and the specifications of the developed BTM-NEU are shown in Fig. 2 and Table 1, respectively. Fig. 2(a) shows the BTM-NEU installed on a sample stage of the Energy Resolved Neutron Imaging System (RADEN) in the MLF, J-PARC. The dimensions and the weight of the BTM-NEU are determined as the BTM-NEU can be securely installed on the sample stage of RADEN with a neutron beam height of 1.773 m from the floor surface. Fig. 2(b) shows clamps placed in the center of the BTM-NEU. A cruciform specimen is fixed by the clamps. The white square area of the figure is an aperture for the passing of a neutron beam. The BTM-NEU can apply biaxial tensile stresses to an ISO-standardized cruciform specimen in horizontal (X) and vertical (Y) directions in any stress ratio. The BTM-NEU can perform three kinds of controls, load control, displacement control, and strain control. The load capacity is 50 kN in each direction. The crosshead speed range is 0.001 to 60 mm/min. The displacement accuracy is less than 1% of the displacement set value of 85 mm. The data sampling rate is 1 to 200 Hz. A cruciform specimen basically conforms to the ISO standard. The total length and the width of the arms of a typical ISO standardized cruciform specimen are 130 mm and 30 mm, respectively. A non-ISO standardized cruciform specimen and a strip form specimen can be also tested, where dimensions of a mountable specimen are as follows: the total length is larger than 130 mm, the width of the arm is smaller than 30 mm, and the thickness is 0.5 to 10 mm. Different clamps are used with 4 mm in thickness as the border.

**Table 1:** Specifications of the BTM-NEU.

Biaxial directions	Horizontal (X) and Vertical (Y) directions
Test types	Biaxial tensile test, Uniaxial tensile test
Control types	Load control, Displacement control, Strain control
Load capacity (kN)	50
Biaxial stress ratios, σ_x_:σ_y_	Any ratio can be set. Typical nine ratio are 1:0, 4:1, 4:2, 4:3, 1:1, 3:4, 2:4, 1:4, 0:1.
Crosshead speed range (mm/min)	0.001–60
Displacement accuracy	≦1% of the displacement set value (85 mm)
Data sampling rate (Hz)	1–200
Dimensions (HxWxD) (mm)	1475 × 1450 × 415
Weight (kg)	1390
Mountable specimen	Shape: ISO standardized cruciform, non-ISO standardized cruciform, strip form, etc. Dimensions (mm): total length ≧130, arm width ≦30, thickness = 0.5–10

The BTM-NEU is controlled by the dedicated system independently of the control and data acquisition system of RADEN because the remote control of the BTM-NEU is prohibited by a copyright of the control software of the BTM-NEU. Therefore, both systems are currently synchronized through an external timer. The anisotropic yielding function of the specimen can be analyzed with another software installed in the control system of the BTM-NEU.

### Experimental performance evaluation

#### Samples

The materials and the mechanical properties of the cruciform specimens for biaxial tensile tests are shown in Table 2. In tensile tests in different stress ratios and a long-time biaxial tensile test, cold-rolled mild steel and hot-rolled high-tensile-strength steel materials were used for the cruciform specimens, respectively, where the cold-rolled mild steel material is a standard material of the research committee for the sophistication of steel sheet forming technology with the aid of advanced multiaxial stress testing methods established in the Iron and Steel Institute of Japan. In a trial measurement of in-situ Bragg-edge neutron imaging, the hot-rolled high-tensile-strength steel materials was used.

**Table 2:** Materials and mechanical properties of the cruciform specimens for biaxial tensile tests.

Test types		Biaxial tensile tests in different stess ratios	Long-time biaxial tensile test and in-situ Bragg-edge neutron imaging measurement
Materials		Cold-rolled mild steel	Hot-rolled high-tensile- strength steel
Mechanical properties	Tensile strength (N/mm^2^)	296	603
	Yield strength (N/mm^2^)	153	463
	Elongation (%)	28	28

**Fig. 3:**
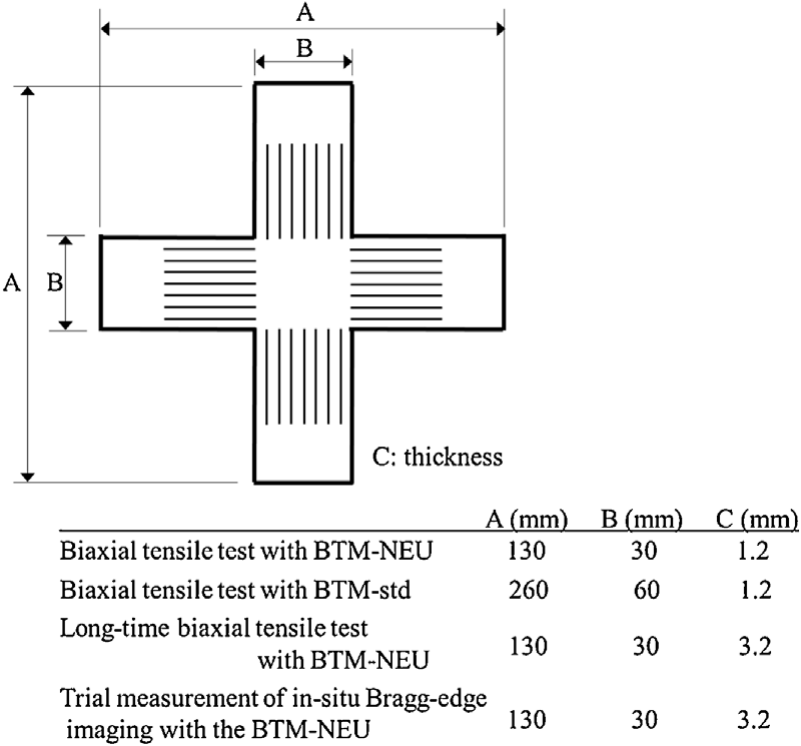
Shapes of cruciform specimens for biaxial tensile tests with the BTM-NEU and the BTM-std, a long-time biaxial tensile test and a trial measurement of in-situ Bragg-edge neutron imaging with the BTM-NEU. A, B and C are the total length, the width and the thickness of the arms of the specimen, respectively. Each arm of the specimen has seven slits as specified in the ISO standard.

The cruciform specimens conform to the ISO standard and their shapes are shown in Fig. 3. A pair of arms of each specimen is parallel to the rolling direction of the rolled steel material. Each arm has seven slits as specified in the ISO standard. In the cases of tensile tests in stress ratios of 1:0 and 0:1, uniaxial specimens were used instead of cruciform specimens. 13B standard uniaxial test specimens of the Japanese Industrial Standards (JIS) Z2201 were used for the tensile tests with the BTM-std. On the other hand, specimens used for the tests with the BTM-NEU were similar to the 13B standard ones but different in curvature radius of the edges of the reduced section. In the case of the ratio of 1:0 the longitudinal direction of the specimen is parallel to the rolling direction of the rolled steel material. On the other hand, in the case of the ratio of 0:1 the transverse direction of the specimen is parallel to the rolling direction of the rolled steel material.

#### Evaluate methods

##### Biaxial tensile tests in different stress ratios

To verify that the performance of the BTM-NEU is equivalent to that of the BTM-std, biaxial tensile tests were performed in nine stress ratios (1:0, 4:1, 2:1, 4:3, 1:1, 3:4, 1:2, 1:4, and 0:1) at a loading rate of 125 N/s (7.5 kN/min) with the BTM-NEU. The rolling directions of the cruciform and uniaxial specimens were fixed so as to be parallel to the X direction of the BTM-NEU and the BTM-std. Two strain gauges (Showa Measuring Instruments Co., Ltd., Y11-MA-1-120-11-P4-L005-BSS) were placed in the X and Y directions at the positions 10.5 mm away from the center of each cruciform specimen used for the tests with the BTM-NEU. On the other hand, a strain gauge (Kyowa Electronic Instruments Co., Ltd., KFGS-1N-120-C1-11) was placed in the tensile direction at the center of each uniaxial specimen used for the tests with the BTM-NEU.

##### Long-time biaxial tensile test

In Bragg-edge neutron imaging experiment, the measurement time necessary for getting data of sufficient accuracy depends on material type and thickness of a specimen, a neutron beam flux, etc. When using a thin steel specimen with several mm in thickness, it is sometimes needed to measure a neutron transmission spectrum for a longer time than 10 h with the current RADEN. Moreover, it is not adequate to attach a strain gauge on the central part of a specimen because the gauge changes a neutron transmission spectrum. Therefore, the BTM-NEU is necessary to be controlled by load during the Bragg-edge neutron imaging experiment. Thus, a long-time biaxial tensile test was performed with the BTM-NEU. The stress ratio was kept to be 1:1 by load control and a loading rate was 333.3 N/s (20 kN/min). The holding times were 20 h for 0 kN (non-loading), 23.5 h for 20 kN, 45 kN, and 0 kN (post-unloading). The total test time was 90.5 h. The rolling direction of the cruciform was fixed so as to be parallel to the X direction of the BTM-NEU.

**Fig. 4:**
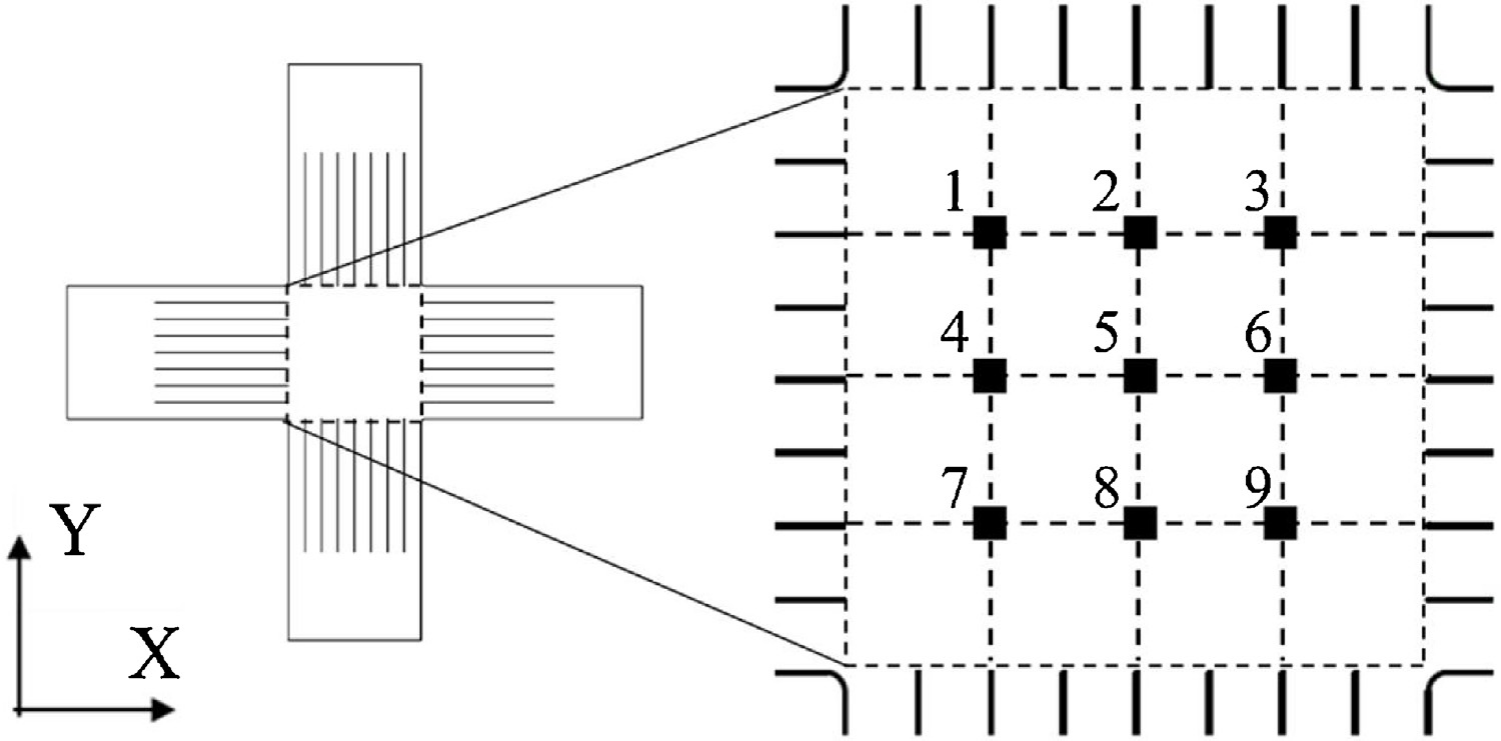
Strain gauge positions (1–9) on the cruciform specimen.

To confirm the homogeneity of strain distribution in the ISO standardized cruciform specimen using the BTM-NEU as it is confirmed using the BTM-std, a strain measurement using strain gages was adopted as a simple method. Considering the sizes of a strain gage and the central part of the specimen, nine strain gauges (Kyowa Electronic Instruments Co., Ltd., KFGS-1N-120-C1-11) were placed in the X direction at 7.5 mm intervals in the central part (30 mm × 30 mm) of the specimen as shown in Fig. 4.

##### Trial measurement of in-situ Bragg-edge neutron imaging

As a trial measurement of in-situ Bragg-edge neutron imaging, the BTM-NEU was installed in RADEN. The central part of the cruciform specimen (30 mm × 30 mm) was perpendicularly irradiated by a pulsed neutron beam while applying biaxial tensile load to the specimen. The proton beam power for neutron production was 150 kW. A stress ratio was 1:1 and a loading rate was 333.3 N/s (20 kN/min). The load conditions were 0 kN (non-loading), 20 kN, 45 kN, and 0 kN (post-unloading). The measurement time, which is equivalent to the holding time, was 5 h for each load condition. The neutron transmission spectrum was measured with a GEM (Gas Electron Multiplier) type neutron area detector and the Bragg-edge of the α-Fe {110} plane was analyzed by the RITS code [15] to determine the strain in the thickness direction of the specimen. Since the neutron beam flux and the detector efficiency were low in this measurement, the neutron transmission spectrum was averaged over the central part of the specimen to make the accuracy of the spectrum sufficient in this paper.

#### Results

##### Biaxial tensile tests in different stress ratios

**Fig. 5:**
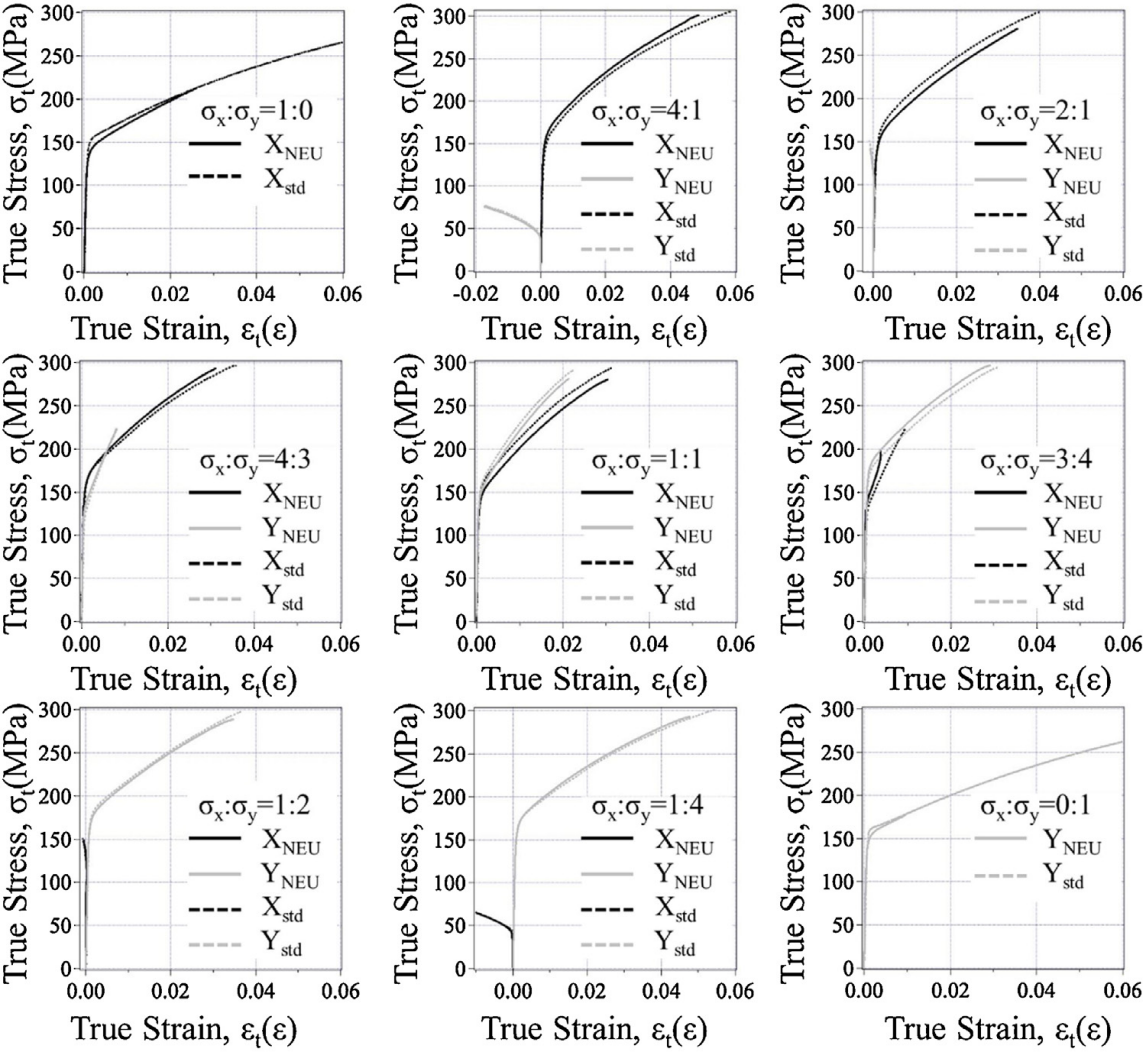
True stress – true strain diagrams obtained by tensile tests under nine biaxial stress ratios. X_NEU_, Y_NEU_, X_std_, Y_std_ are stresses in the X and Y directions obtained with the BTM-NEU and the BTM-std, respectively.

Fig. 5 shows true stress-true strain curves obtained in biaxial tensile tests in different stress ratios with the BTM-NEU and the BTM-std. The true stress σ_t_ and the true strain ε_t_ were calculated on the formulas σ_n_(1 + ε_n_) and ln(1 + ε_n_), respectively, where σ_n_ is the nominal stress expressed by σ_n_ = P/A_0_ with the load P acting on the cross-sectional area A_0_, and ε_n_ is the nominal strain which is equivalent to the engineering strain ε.

In each stress ratio the curve obtained with the BTM-NEU agrees very well with that obtained with the BTM-std except for plastic region. Although there is no applicable difference in the curves in plastic region for the stress ratios of 1:2 and 1:4, small difference in the curves in plastic region is recognized for the stress ratios of 1:0, 4:1, 2:1, 4:3, 1:1, 3:4, and 0:1. The small difference may not be fundamental and may depend on the material of the specimen because it is probable that the mechanical properties of a cold-rolled steel plate slightly vary in the direction perpendicular to the rolled direction of the plate. These things suggest that the BTM-NEU has reliability comparable to that of the BTM-std.

##### Long-time biaxial tensile test

**Fig. 6:**
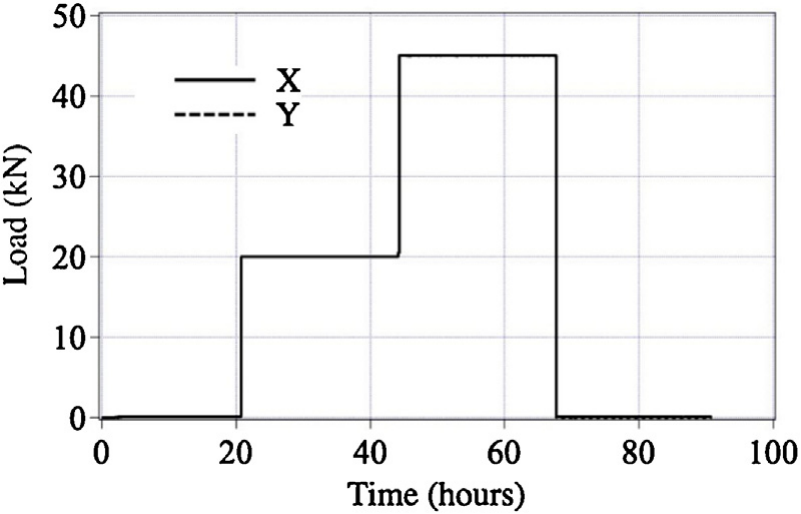
Change of load during a long-time biaxial tensile test.

Fig. 6 shows changes of loads in the X and Y directions during the long-time biaxial tensile test. There is no appreciable load fluctuation and difference in loads in the X and Y directions. Table 3 shows the mean values and the standard deviations of values of the nine strain gauges in four load conditions. The ratio of the standard deviation to the mean value is less than 3% except for 0 kN (non-loading). These results show that the BTM-NEU has excellent engineering reliability and stability even during a tensile test with a time of 20 h or more.

**Table 3:** Mean values and standard deviations of strains in the central part of the cruciform specimen during a long-time biaxial tensile test.

Load (kN)	0 (Non-loading)	20	45	0 (Post-unloading)
Mean value of strain (με)	1.8	787.1	11166.7	9495.3
Standard deviation of strain (με)	4.2	23.7	281.5	283.6

##### Trial measurement of in-situ Bragg-edge neutron imaging

**Fig. 7:**
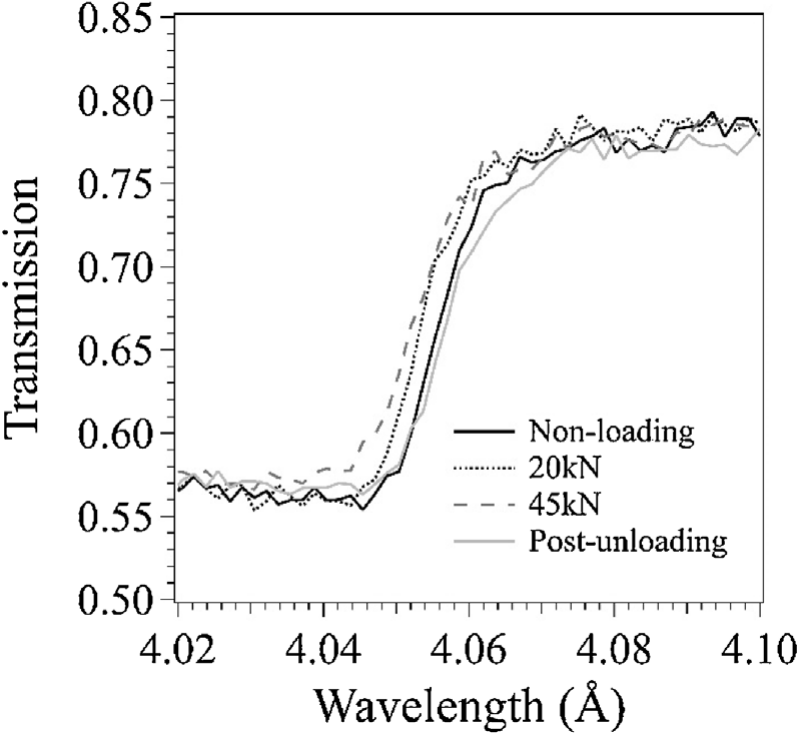
Neutron transmission spectra near the α-Fe {110} Bragg-edge averaged over the central part of the cruciform specimen.

**Fig. 8:**
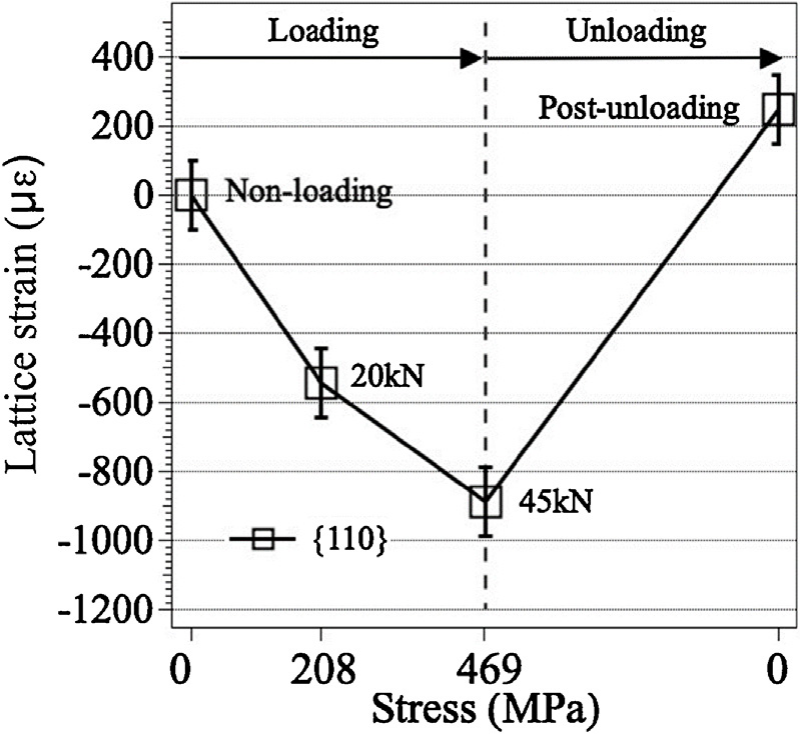
Stress dependence of lattice strain obtained from the shift of the α-Fe {110} Bragg-edge.

Fig. 7 shows the neutron transmission spectra near the α-Fe {110} Bragg-edge averaged over the central part of the cruciform specimen. The spectra show clear Bragg-edges. The shift of the position and the change of shape of the Bragg-edge are observed by changing the loading conditions, 20 kN, 45 kN, and 0 kN (post-unloading). Fig. 8 shows stress dependence of lattice strain obtained from the shift of the Bragg-edge. The lattice strain is an averaged value in the thickness direction of the specimen. The strain indicates the compression of the specimen along the thickness direction by applying tensile loads in the X and Y directions of the cruciform specimen, and the compressive strain increases with increasing loads. In the post-unloading condition, residual strain is observed. From these results, it is confirmed that the BTM-NEU can be applied to pulsed neutron experiments using a Bragg-edge imaging method. Detailed analysis of the spatial distribution of crystallographic information of the specimen will be possible by future increase of neutron beam flux in the MLF of the J-PARC and upgrading the neutron area detector and the analysis method.

### Summary

We developed a biaxial tensile testing machine for pulsed neutron experiments (BTM-NEU), which can test an ISO-standardized cruciform specimen as the BTM-std. Although the specifications of the BTM-NEU are different from those of the BTM-std, it was experimentally confirmed that the performance of the BTM-NEU is equivalent to that of the BTM-std and the BTM-NEU has also excellent long-time reliability and stability which is necessary for pulsed neutron experiments. Moreover, the BTM-NEU can be applied to pulsed neutron experiments using a Bragg-edge imaging method. These results mean that the microscopic information of specimens obtained by pulsed neutron experiments with the BTM-NEU can be directly correlated with the macroscopic information of specimens obtained with the BTM-std. For example, strain in the thickness direction of the plate obtained by the Bragg-edge imaging neutron imaging measurement can be provided as an additional parameter used in a simulation of the flange forming, which is considered to be changed by the strain in the thickness direction due to the increase or the decrease in thickness of the plate. If spatial distribution of microscopic information such as phase, texture, crystal size, dislocation density, etc. can be measured by the Bragg-edge neutron imaging measurement, the microscopic information would be useful to understand the mechanism of differential work hardening seen in contours of plastic work obtained by biaxial tensile tests with the BTM-std. By the progress of such verification research, the microscopic information obtained by neutron experiments with the BTM-NEU is expected to contribute to further improvement of the elastoplastic analysis model of functional thin steels used for press forming.
